# Morphological, Physiological, Biochemical, and Molecular Characterization of Fungal Species Associated with Papaya Rot in Cameroon

**DOI:** 10.3390/jof11050385

**Published:** 2025-05-17

**Authors:** Moussango Victor Davy, Voundi Olugu Steve, Tchabong Raymond Sammuel, Marie Ampères Bedine Boat, Ntah Ayong Moise, Anna Cazanevscaia Busuioc, Priscile Ebong Mbondi, Andreea Veronica Dediu Botezatu, Manz Koule Jules, Maria Daniela Ionica Mihaila, Rodica Mihaela Dinica, Sameza Modeste Lambert

**Affiliations:** 1 Biotechnologies Laboratory, University Institute of Technology, University of Douala, Douala 8698, Cameroon; voundisteve@yahoo.fr; 2 Laboratory of Biochemistry, Faculty of Science, University of Douala, Douala 24157, Cameroon; ntamoise@gmail.com (N.A.M.); priscilleebong91@gmail.com (P.E.M.); manz2013@yahoo.fr (M.K.J.); samezamste@yahoo.com (S.M.L.); 3 Department of Chemistry, Physics and Environment, Faculty of Sciences and Environment, ‘Dunărea de Jos’ University, 47 Domneasca Str., 800008 Galati, Romania; anna.cazanevscaia@ugal.ro (A.C.B.); andreea.botezatu@ugal.ro (A.V.D.B.); maria.mihaila@ugal.ro (M.D.I.M.); 4 Department of Processing and Quality Control of Aquatic Products, Institute of Fisheries and Aquatic Sciences, University of Douala, Douala 7236, Cameroon; tchabongraymond@yahoo.fr; 5 Phytopathology and Agricultural Zoology Research Unit, Faculty of Agronomy and Agronomic Sciences, University of Dschang, Dschang 67, Cameroon; bedineboat@yahoo.fr

**Keywords:** *Carica papaya* rot, fungi characterization, phylogenetic analysis, pathogenicity, hydrolytic enzymes

## Abstract

Post-harvest decay of *Carica papaya* L. is the primary cause of deterioration in papaya quality and the low economic impact of this sector in Cameroon. Field surveys conducted by teams from the Ministry of Agriculture and Rural Development (MINADER) in Cameroon have primarily associated these decays with fungal attacks. However, to date, no methodological analysis has been conducted on the identification of these fungal agents. To reduce post-harvest losses, rapid detection of diseases is crucial for the application of effective management strategies. This study sought to identify the fungal agents associated with post-harvest decay of papaya *cv* Sunrise solo in Cameroon and to determine their physiological and biochemical growth characteristics. Isolation and pathogenicity tests were performed according to Koch’s postulate. Molecular identification of isolates was achieved by amplification and sequencing of the ITS1 and ITS4 regions. Phylogenetic analysis was based on the substitution models corresponding to each fungal genus determined by jModeltest, according to the Akaike information criterion (AIC). Fungal explants of each identified species were subjected to variations in temperature, pH, water activity, and NaCl concentration. The ability to secrete hydrolytic enzymes was determined on specific media such as skimmed milk agar for protease, peptone agar for lipase, and carboxymethylcellulose for cellulase. These experiments allowed the identification of three fungi responsible for papaya fruit decay, namely *Colletotrichum gloeosporioides*, *Fusarium equiseti*, and *Lasiodiplodia theobromae*. All three pathogens had maximum mycelial growth at a temperature of 25 ± 2 °C, pH 6.5, NaCl concentration of 100 µM, and water activity (aw) equal to 0.98. The three fungal agents demonstrated a strong potential for secreting cellulases, lipases, and proteases, which they use as lytic enzymes to degrade papaya tissues. The relative enzymatic activity varied depending on the fungal pathogen as well as the type of enzyme secreted. This study is the first report of *F. equiseti* as a causal agent of papaya fruit decay in Cameroon.

## 1. Introduction

Papaya (*Carica papaya* L.) is a widely cultivated tropical fruit in Cameroon and the most sought after in the market, due to its high content of minerals and vitamins. It is strongly recommended for its immunostimulant properties [1,2,3]. Papaya is the fourth most produced tropical fruit in the world, after banana, orange, and mango [4,5]. Their production in Africa is estimated at 7,369,579 tonnes, of which 43,000 tons are from Cameroon [6].

Despite its economic, nutritional, and medicinal importance, the production and marketing of papaya are hindered by pests and diseases, especially fungal pathogens, particularly those caused by fungal phytopathogens. The most common fungal diseases affecting papaya worldwide are caused by fungi of the genera *Colletotrichum*, *Rhizopus*, *Fusarium*, *Ascochyta*, *Cladosporium*, *Alternaria*, and many others [7,8,9,10]. These fungi colonize the fruit tissues during the growth and development phase, remaining endophytic without producing symptoms until the fruit ripens. As the fruit ripens, several biochemical and physiological changes occur, including the production of ethylene, the accumulation of soluble sugars, and a decrease in the concentrations of phytoalexins [11,12,13]. These modifications weaken the fruit’s ability to activate its natural defenses [14]. In response to these biochemical changes, the phytopathogenic fungi transit from an endophytic state to a necrotrophic state. This characteristic complicates the early detection of diseases and the prevention of damage caused by these phytopathogens.

Numerous studies have identified *Colletotrichum gloeosporioides* as the primary causal agent of papaya anthracnose [1,12,15]. This pathology is quiescent, and typical symptoms are generally not visible in the field during papaya growth. However, it manifests post-harvest, as fruits begin to ripen during transportation within the supply chain [16]. Furthermore, previous studies conducted in Brazil, India, Malaysia, and Nigeria have shown that inexperienced handling of papayas leads to lesions on the fruits during the post-harvest agricultural chain and promotes the development of wilt fusariosis caused by *Fusarium* spp. (*F. solani*, *F. semitectum*, *F. oxysporum*, *F. acuminatum*, *F. chlamydosporum* and *F. equiseti*) [17,18,19,20]. *Fusarium* species are considered secondary pathogens because they require preliminary stress (biotic or abiotic) to induce infection in fruits. They persist in the internal tissues of fruit and manifest late on the fruit surface as a white and compact mycelium [21]. *Fusarium solani* is the most widespread and frequently reported *Fusarium* species capable of causing papaya fruit rot. However, *F. equiseti* was first reported as a pathogen of papayas in Malaysia [22]. *F. solani* is also considered a rot agent of watermelon, pepper, and wheat [18,23,24]. Other reports on pathological rot of papaya fruits have been documented in Mexico, China, Ghana, and Ethiopia by researchers who associated the species *Lasiodiplodia theobromae* with these losses [25,26]. This fungus can infect papayas by causing excessive softening of the fruits, the development of dark lesions, and mummification of the infected area post-harvest [27].

These pathogenic fungi infect papayas and alter their organoleptic and nutritional properties. In Sub-Saharan Africa and particularly in Cameroon, fungal fruit rots are the most devastating and lead to a significant decrease in the quality and quantity of papayas. For many local farmers, fungal diseases are the primary cause of reduced production yields (60–100%) [16,28]. Moreover, the toxins secreted and dispersed by these fungi in papayas are dangerous to consumers. For example, colletotrichum lactones (A-D), lasiodiplodine, melleins, deoxynivalenol (DON), trichothecenes (T-2, HT-2), zearalenone (ZEN), and fumonisins, can lead to food poisoning, immunosuppression, and gastrointestinal disorders in humans when present in high concentrations in the fruits [29,30,31]. Diseases caused by phytopathogenic fungi represent a limit for papaya exports because fruit characteristics, such as the absence of rot signs, are a major requirement to ensure sale on the international market [32,33,34].

To contain this threat, Cameroonian farmers, who have no information about the identity of the pathogens responsible for the rotting of papaya fruits, are using chemical pesticides. The irrational use of these chemical products represents a real and significant risk to the environment and a threat to biodiversity. The severity of diseases induced by these fungi underscores the urgent need for molecular identification and analysis methods of physiological growth parameters. This study aims to identify fungal pathogens associated with post-harvest papaya rot in Cameroon, determine their environmental growth parameters, and characterize their biochemical pathogenic properties.

## 2. Materials and Methods

### 2.1. Collection of Healthy and Infected Papayas

To investigate fungal pathogens present in the most representative papaya storage in Cameroon, all papayas of this study originated from the Njombé-Penja locality, the main papaya production basin of the country, and belonged to the *Sunrise solo* hybrid variety (commercial cultivar). A total of 82 papayas were collected, including 42 fruits showing symptoms of various rots observable through necrosis or spots (collected for isolation purposes) and 40 apparently healthy papayas (collected for pathogenicity testing). The papaya samples were individually introduced in sterile plastic bags (Whirl-Pak^®^ code 200363, 19 × 38 cm, Pleasant Prairie, WI, USA) and transported to the laboratory.

### 2.2. Isolation of Pathogenic Fungi from Papaya Fruit

Papayas exhibiting rot symptoms were rinsed under running water to remove solid debris. Each papaya was surface-sterilized with 70% ethanol for 2 min, followed by three sequential rinses with sterile distilled water (SDW). Using sterilized forceps, 5 mm tissue fragments were excised from the lesion margins and aseptically transferred to potato dextrose agar (PDA) plates supplemented with ampicillin and penicillin (175 ppm each). Plates were incubated at 25 ± 2 °C and monitored daily. Morphologically distinct fungal colonies were subcultured onto fresh PDA medium. Three successive subcultures were performed to obtain pure isolates [35].

### 2.3. Pathogenicity Test

To confirm that the isolated fungi were indeed responsible for the observed rot on healthy papayas cv. Sunrise solo during storage, a pathogenicity test was conducted by direct inoculation of fungal conidia of the seven isolates obtained from papaya fruits according to the protocol described by Anthony et al. [36] and modified by Vu et al. [37].

The conidial suspension was prepared from 8-day-old fungal cultures on PDA. Briefly, 100 μL of sterile distilled water (SDW) was added to the surface of the cultures and scraped superficially with a sterile loop. The resulting conidial solutions were filtered and stored separately. The papaya fruit cv Sunrise solo was washed under running water, then surface-sterilized with 70% ethanol for 1 min and 1% NaOCl for 1 min, followed by three consecutive rinses with SDW. The papayas were dried, and then artificial wounds (7 mm in length and 4 mm in depth) were created on the lateral surface of the papayas using a sterile scalpel. After, a volume of 10 μL from the conidial suspension from each isolate, calibrated to 10^6^ conidia/mL, was individually introduced into wounds of each papaya fruit. The artificial wounds were covered with small fragments previously taken from the papayas. Five repetitions (trials) were carried out for each isolate. For the control, five papayas were inoculated with SDW under the same conditions as the infected papayas. To control a potential cross-contamination, after each inoculation with a conidial solution from a specific isolate, the work surface was disinfected, and tools were flame-sterilized between samples. The inoculated papayas were incubated individually in sterile plastic containers lined with moist paper towels at 25 ± 2 °C and observed daily for a period of 12 days. The pathogenicity of the isolates was confirmed by observing lesions and necrosis on the papaya surface. The diameter and depth of the necrosis were measured. Finally, re-isolations were performed from the inoculated fruits to fulfill Koch’s postulates.

### 2.4. Morphological Characterization of Fungi

Identification of the three pathogenic isolates (DMV2, DMV3, and DMV5) was based on macroscopic and microscopic observations. At the macroscopic level, we relied on colony shape, appearance, and growth rate. Microscopic observations of 6-day-old fungal isolates incubated at 25 ± 2 °C were made by spreading a suspension containing conidia and mycelial filaments between the slide and coverslip. These observations were made with a light microscope at 40× (Olympus CX21i microscope, Tokyo, Japan). They focused on conidial shape, size, and conidial load. The morphological characteristics of the different isolates were compared with those found in the databases to identify the genus to which they were related [38,39].

### 2.5. Molecular Identification of Fungi

#### 2.5.1. DNA Extraction and Purification

Genomic DNA from three pathogenic isolates was extracted from 200 mg of mycelial mat taken from a 7-day culture grown on PDA using the NucleoSpin^®^ genomic DNA isolation kit, Plant II (Macherey-Nagel, Düren, Germany) according to the manufacturer’s instructions. DNA purity was assessed using a Nano Photometer P300 (Implen; Microvolume Spectrophotometer, München, Germany). For quantification, 3 µL of genomic DNA was pipetted and the optical density measured at 260 and 280 nm directly in the drop.

#### 2.5.2. PCR Amplification

This experiment consisted of amplifying, by the PCR technique, the internal regions transcribed by ITS1 and ITS4 (Table 1), synthesized by Oligo Express. The choice of internal transcribed regions in this primary molecular analysis is due to the fact that ISTs are conserved regions of DNA that have remained unchanged and have not undergone any modification during evolution.

In a 0.5 mL tube containing 45 µL of a mixture (5 µL of buffer, 0,4 µL of 10 mM dNTPS, 2.5 µL ITS1 primer, 2.5 µL ITS4 primer, 0.25 µL Taq polymerase (5U/µL), and 34.35 µL SDW), 5 µL genomic DNA (5 to 40 ng) was added. The amplification reaction was carried out in a Techne Progene FPR0G050 DNA Thermal Cycler (Thermocycler Techne, Morancé, France) according to the program comprising a denaturation step at 94 °C for 3 min, followed by 35 cycles at 94 °C for 30 s, at 50 °C for 30 s, at 72 °C for 40 s, and finally the final extension was carried out at 72 °C for 5 min.

#### 2.5.3. Amplification on Agarose Gel Electrophoresis

PCR products were electrophoresed on a 1% agarose gel in tris-acetate EDTA (TAE). PCR products were spiked with a deposition solution (0.25% bromophenol; 0.25% xylene cyanol; 25 mM EDTA; 50% glycerol). Migration took place in TAE buffer (1.6 mM Tris-HCl; 1.6 mM sodium acetate; 0.04 mM EDTA; pH 8.0) at 120 volts. After migration, the gel was soaked in ethidium bromide solution (0.5%) for 10 min, then rinsed with water for 15 min and placed under a UV lamp to visualize and photograph the bands.

#### 2.5.4. Sequencing

PCR amplification products were sequenced using an automated DNA sequence analysis system (Beckman Coulter Capillary Sequencer 2000 XL, Krefeld, Germany) for 15 h, and the raw data were then analyzed using CEQ XL version 4.3 software. The resulting sequences were subjected to a sequence homology search using the BLAST (Basic Local Alignment Search Tool) program on the NCBI (National Center for Biotechnology Information) website to search for the closest sequences deposited in GenBank (https://blast.ncbi.nlm.nih.gov/Blast.cgi (accessed on 8 November 2020)) [41]. The sequences derived in this study were deposited in GenBank, and the following accession numbers were obtained: PV576402, PV576403, and PV576404 for DMV2, DMV3, and DMV5, respectively.

#### 2.5.5. Phylogenetic Analysis

The sequences generated in this study were compared to those referenced in GenBank using the BLASTn tool. A total of 120 sequences belonging to the same genera as our sequences (40 per fungal genus × 3) were used. All species corresponding to the best hits on GenBank, along with their accession numbers and base pair counts, are listed in Table S1. DNA sequences of each fungal genus were aligned using BioEdit 7.1.3 and manually adjusted to maximize alignment and minimize gaps. All ambiguous positions were removed for each sequence pair, and genetic distances were calculated [42]. Substitution models corresponding to each fungal genus in our dataset were determined by jModeltest v. 2.1.10 under the Akaike Information Criterion (AIC). Maximum likelihood (ML) analysis was performed for each fungal genus using slow bootstrap with 1000 replicates in MEGA XI [43]. The evolutionary models used to construct the ML phylogenetic trees were Tamura-Pei-Model 2 with Gamma distribution (TPM2+G) for the *Colletotrichum* genus, Tamura-Pei-Model 3 with Gamma distribution (TPM3+G) for the *Fusarium* genus, and Kimura 2-Parameter (K80) for the *Lasiodiplodia* genus. For the different genera in our dataset, the *Phytophthora infestans* (OR145849.1) species was used as the outgroup.

### 2.6. Influence of Environmental Parameters on Mycelial Growth

#### 2.6.1. Effect of Temperature on Mycelial Growth

The effect of temperature on the mycelial growth of papaya pathogenic fungi was determined according to the protocol used by Compaoré et al. [44]. Specifically, mycelial disks (5 mm diameter) from a 2-day culture of each isolate were placed in the center of Petri dishes containing 10 mL of PDA medium and then incubated at different temperatures, 15 °C, 20 °C, 25 °C, 30 °C, 35 °C, and 40 °C. For each isolate and incubation temperature, the tests were repeated three times. The radial growth of each fungus was observed daily over 3 days for the DMV5 isolate and 7 days for DMV2 and DMV3 isolates. The diameters of growth were measured on the last day, and the mean diameter of mycelial growth at a precise temperature (DM_T_o) was calculated according to Formula (1) below, expressed in mm/day.

DM_T_° = dh_T_° + dv_T_°/2 (1)

DM_T_°: Mean diameter of mycelial growth at precise temperature; dh_T_°: horizontal mycelial diameter at precise temperature; dv_T_°: vertical diameter of mycelium at precise temperature.

#### 2.6.2. Effect of pH on Mycelial Growth

The influence of pH on the mycelial growth of papaya pathogenic fungi was determined according to the protocol used by Kannan and Dhivya [45] as well as Ahohuendo et al. [46]. This consisted of preparing 10 mL of PDA culture medium adjusted to different pH levels: 4.5, 5.5, 6.5, 7.5, 8.5, and 9.5 by using 1N HCl or 1N NaOH with a digital pH meter (Hand Held TDS Meter, EZ-9902 Yinmik, Jinan, China). Then, mycelial disks (5 mm) were removed from a 2-day-old culture using a sterile cookie cutter, placed in the center of Petri dishes, and incubated at 25 ± 2 °C for 3 days for DMV5 isolate and 7 days for DMV2 and DMV3 isolates. Growth diameters were measured on the last day, and the mean diameter of mycelial growth at a precise pH (DMpH) was calculated according to Formula (2) below, expressed in mm/day.

DMpH = (dhpH + dvpH)/2(2)

DMpH: Mean mycelial growth diameter at precise pH; dhpH: horizontal mycelial diameter at precise pH; dvpH: vertical mycelial diameter at precise pH.

#### 2.6.3. Effect of Salt Stress on Mycelial Growth

The effect of NaCl concentration on pathogen growth was investigated on PDA medium prepared at various NaCl concentrations: 100 µM, 250 µM, 500 µM, 750 µM, 1000 µM, and 1250 µM [47]. Mycelial disks (5 mm) were removed from a 2-day-old culture using a sterile cookie cutter, placed in the center of Petri dishes, and incubated at 25 ± 2 °C for 3 days for DMV5 isolate and 7 days for DMV2 and DMV3 isolates. Growth diameters were measured on the last day, and the mean diameter of mycelial growth at a precise NaCl molar concentration (DM_[NaCl]_) was calculated according to Formula (3) below, expressed in mm/day.

DM_[NaCl]_ = (dh_[NaCl]_ + dv_[NaCl]_)/2 (3)

DM_[NaCl]_: Mean mycelial growth diameter at precise [NaCl]; dh_[NaOH]_: horizontal mycelial diameter at precise [NaCl]; dv_[NaCl]_: vertical mycelial diameter at precise [NaCl].

#### 2.6.4. Effect of Water Potential on Mycelial Growth

The influence of water potential on the mycelial growth of papaya pathogenic fungi was evaluated on PDA medium (0, 995 a_w_) modified by adding increasing amounts of glycerol to obtain media with water activities of 0.88 a_w_, 0.90 a_w_, 0.92 a_w_, 0.94 a_w_, 0.96 a_w_, and 0.98 aw according to the revised protocol of Jabiri et al. [48]. The water activity of the different media was measured with an AW meter (HC2A-AW, Rotronic, Switzerland). Mycelial disks (5 mm) were removed from a 2-day-old culture using a sterile punch, placed in the center of Petri dishes, and incubated at 25 ± 2 °C for 3 days for the DMV5 isolate and 7 days for the DMV2 and DMV3 isolates, respectively. Growth diameters were measured on the last day, and the mean diameter of mycelial growth at precise aw (DMa_w_) was calculated according to Formula (4) below, expressed in mm/day.

DMa_w_ = (dha_w_ + dva_w_)/2 (4)

DMa_w_: average mycelial growth diameter at precise a_w_; dha_w_: horizontal mycelial diameter at precise a_w_; dva_w_: vertical mycelial diameter at precise a_w_.

### 2.7. Biochemical Characterization: Highlighting of Hydrolytic Enzymes Produced by Fungi

- Production of protease

The protease production capacity was determined by transferring the obtained fungi to a 40% skimmed milk agar medium [49,50]. This medium allows the demonstration of proteolytic activity by the appearance of a clear halo around the producing colony. To better observe the microbial growth, the milk agar medium was supplemented with 2% Coomassie blue, and the fungal explants of the various pathogens were placed in the center of Petri dishes. The negative control was made up of a milk-free medium consisting solely of agar and Coomassie. The tests were performed in triplicate for all identified fungi, and Petri dishes were stored for 3 days at 25 ± 2 °C. The determination of the relative protease activity (RPA) was made by measuring the diameters of the formed rings by using a slide gauge.

- Production of lipase

The ability of fungi to produce lipases was demonstrated by subculturing fungal explants on solid peptone agar medium supplemented with 0.01% phenol red, 1.0% olive oil, 10 mM CaCl_2_, pH 7.3 [51,52]. The negative control consisted of the same culture medium without olive oil. For each fungus, tests were run three times and incubated at 25 ± 2 °C for 3 days. The color change from red to yellow around the growth indicates the production of lipase. The tests were carried out in triplicate for each identified fungal species. Relative lipase activity (RLA) was determined by measuring the diameter of the growth halo that was formed around the fungal explant by using a slide gauge.

- Cellulase production

The cellulolytic activity of fungi was demonstrated on medium (pH 7.0) consisted of NaCl (0.5 g × L^−1^), H_2_PO_4_ (1.0 g × L^−1^), MgSO_4_ × 7H_2_O (0.5 g × L^−1^), MnSO_4_×H_2_O (0.01 g × L^−1^), NH_4_NO_3_ (0.3 g × L^−1^), FeSO_4_ × 7H_2_O (0.01 g × L^−1^), carboxymethylcellulose (10 g × L^−1^), and agar (15 g × L^−1^). After the growth period (2 days), the dishes were stained with a Congo red solution (0.1%), which binds selectively to the cellulose polymers. The dishes were then washed with a NaCl solution (1 M) for 1 h [51,53,54]. The negative control consisted of the same culture medium without carboxymethylcellulose. The test was performed in triplicate for each identified fungal species, and Petri dishes were stored for 3 days at 25 ± 2 °C. Relative cellulase activity was determined by measuring the diameter of the formed rings. The result of the relative cellulase activity (RCA) was obtained by measuring the diameters (in cm) of the formed rings (growth aureoles) by using a slide gauge.

### 2.8. Statistical Analysis

The data on mycelial growth measurements under the effect of environmental conditions (temperature, pH, [NaOH], and aw) were analyzed using SPSS software version 16.0. A homogeneity of variance test was performed to assess the equality of variances between groups. Subsequently, ANOVA was used to compare means among different groups, and Tukey’s test was conducted for pairwise comparisons. The results were applied to mean diameters (±SD) from triplicate experiments and presented in graphical form (using GraphPad software, version 10.4.0), with the significance level set at *p* < 0.05 for each test. Phylogenetic analysis of DNA sequences was performed using the Clustal W program of the MEGA-XI software package (Molecular Evolutionary Genetics Analysis, https://www.megasoftware.net/, accessed on 14 May 2025), as described in Section 2.5.5.

## 3. Results

### 3.1. Fungal Isolates and Rot Symptoms on Papayas

**Figure 1:**
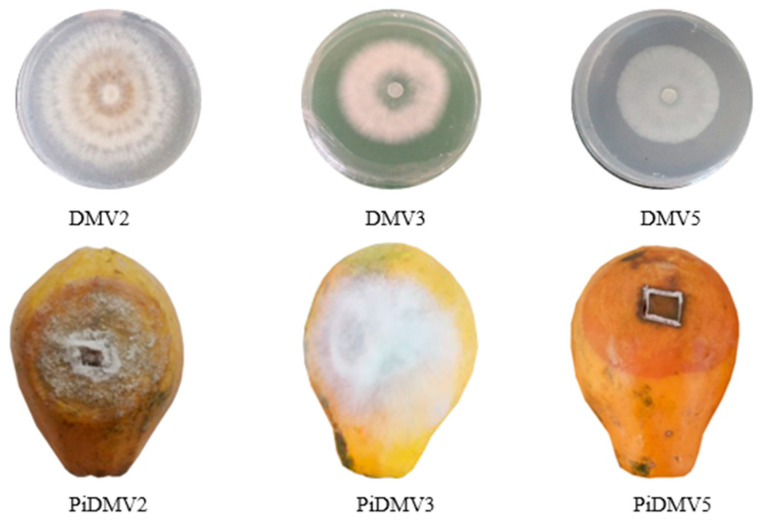
Pathogenic isolates from papayas on PDA and papayas infected with the isolates. DMV2, DMV3, and DMV5 for isolate 2, 3, and 5. PiDMV2, PiDMV3, and PiDMV5 for papayas infected with isolates DMV2, DMV3, and DMV5, respectively.

Seven fungal isolates, designated as DMV1, DMV2, DMV3, DMV4, DMV5, DMV6, and DMV7, were successfully isolated and cultured on PDA from papaya fruits of the Sunrise solo cultivar displaying symptoms of rot. These isolates exhibited distinct cultural characteristics, including variable growth rates. To verify their pathogenicity, the isolates were subjected to artificial inoculation tests on papayas. The results indicated that only DMV2, DMV3, and DMV5 isolates were pathogenic, each inducing a specific type of rot on healthy papayas within 10 days. The outcomes of the pathogenicity assays were illustrated in Figure 1.

**Figure 2:**
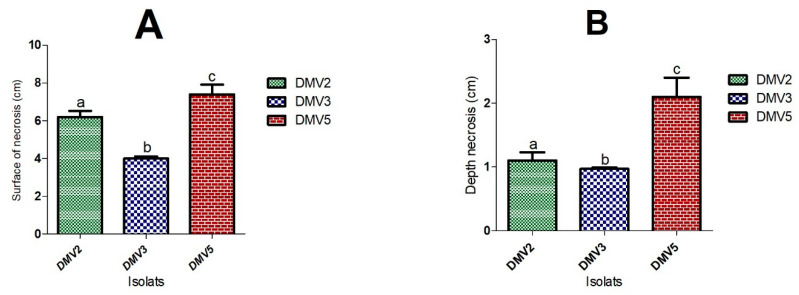
Variation in necrosis induced by pathogenic fungal isolates from papaya. (**A**) Surface area. (**B**) Depth. Three independent experiments were performed in triplicate. Bars represent the standard error. The histograms bearing the different letters (a, b, and c) are significantly different according to Tukey’s statistical test at *p* ≤ 0.05.

The DMV2 isolate induced a grayish brown rot (PiDMV2) on healthy papayas, similar to anthracnose. The DMV3 isolate caused a white and cottony rot (PiDMV3) on healthy papayas, similar to fusariosis. The DMV5 isolate induced a soft and transparent rot (PiDMV5) on healthy papayas. These symptoms were similar to those previously observed on naturally infected papayas during storage. No rot symptoms were observed on the control papayas treated with SDW. The rot symptoms, as well as the measurements of the surface and depth of necrosis, were presented in Table 2 and Figure 2.

After 10 days of incubation, DMV5 isolate was proved to be the most virulent with a rot surface of 7.4 cm and a depth of 2.1 cm, followed by DMV2 isolate with a rot surface of 6.2 cm and a depth of 1.1 cm, and DMV3 isolate with a rot surface and depth of 4.0 cm and 0.97 cm, respectively.

### 3.2. Phenotypic and Morphological Characteristics of Isolates

**Figure 3:**
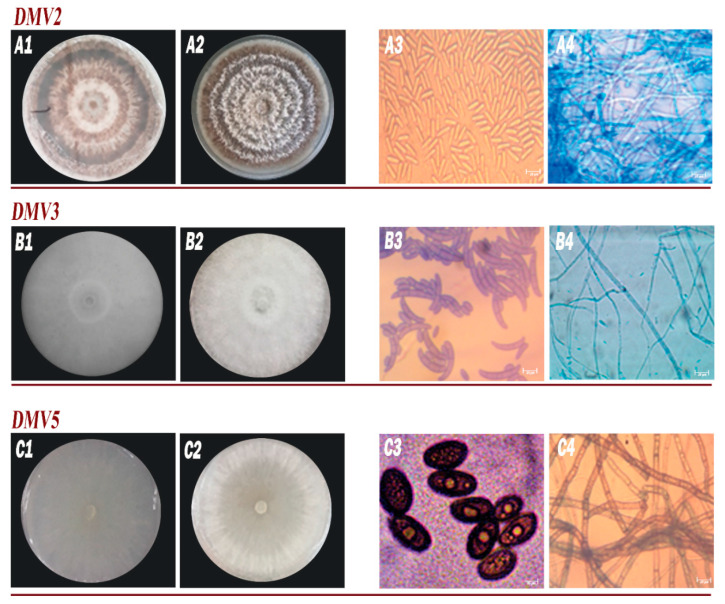
Macroscopic and microscopic aspects of isolates. DMV**2** isolate in PDA medium after 7 days [(**A1**) (front), (**A2**) (reverse), (**A3**) (conidia), (**A4**) (mycelium)]; DMV3 isolate in PDA medium after 7 days [(**B1**) (front), (**B2**) (reverse), (**B3**) (conidia), (**B4**) (mycelium)]; DMV5 isolate in PDA after 3 days [(**C1**) (front), (**C2**) (reverse), (**C3**) (conidia), (**C4**) (mycelium)].

The phenotypic/morphological characteristics of DMV2, DMV3, and DMV5 isolates were shown in Figure 3 and Table 3. These observations revealed a diversity of morphological traits among the isolates.

Isolate DMV2 exhibited white mycelium transitioning to orange-brown, circular colony morphology with convex elevation, a growth rate of 14.1 mm/day, undulated margins, non-septate hyphae, and elongated conidia averaging 14.3 μm in size. These characteristics aligned with the genus *Colletotrichum*.

Isolate DMV3 displayed white mycelium with cottony texture, undulated margins, domed elevation, a growth rate of 13 mm/day, septate hyphae, and fusiform conidia averaging 18.3 μm in size. These features corresponded to the genus *Fusarium*.

Isolate DMV5 showed white mycelium progressing to gray-black, velvety texture, a rapid growth rate of 44 mm/day, flat elevation, septate hyphae, and ovoid conidia averaging 22 μm in size. These traits were consistent with the fungal genus *Lasiodiplodia*.

### 3.3. Molecular Identification and Phylogenetic Analysis

The three isolates, previously characterized through morphological observations (*Colletotrichum*, *Fusarium*, and *Lasiodiplodia*), were subjected to molecular identification. Their genomic DNA was amplified using ITS1 and ITS4 primers, targeting the 18S rRNA subunit of each isolate. Following electrophoresis, the migration observed on the gel showed DNA bands migrating in the 580 bp region for the DMV2 isolate, and 555 bp for DMV3 and DMV5 isolates.

Analysis of the DNA sequences encoding rRNA using the GenBank BLAST tool from NCBI (National Center for Biotechnology Information) allowed the assignment of DMV2, DMV3, and DMV5 isolates to three distinct fungal species. Coverage, accession codes, base pair numbers, similarity percentages with the closest registered strains, and identified species names were detailed in Table 4.

All coverage values with the reference strains listed on NCBI were greater than or equal to 95%. The DMV2 isolate (genus *Colletotrichum*) showed a 100% similarity with FJ940734 and KJ632401 reference strains, both corresponding to *C. gloeosporioides* species. The DMV3 isolate (genus *Fusarium*) showed a 99.46% similarity with MT447515 and KM246255 reference strains, corresponding to *F. equiseti* species. The DMV5 isolate (genus *Lasiodiplodia*) showed similarities of 99.82% and 99.64% with MH049696 and MN180881 reference strains, respectively, identified as *L. theobromae* species.

**Figure 4:**
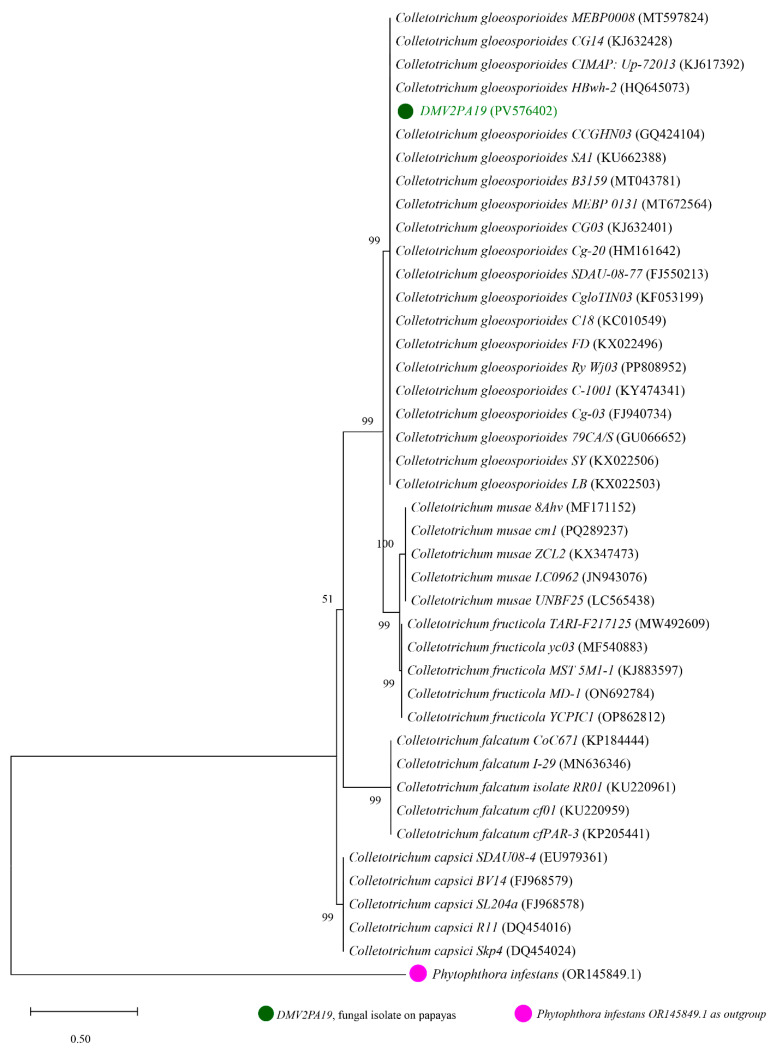
Phylogenetic tree generated from sequences of DMV2 isolate and other reference strains of *Colletotrichum* spp. with *Phytophthora infestans* (OR145849.1) used as an outgroup. The DMV2 isolate from this study is shown in green.

**Figure 5:**
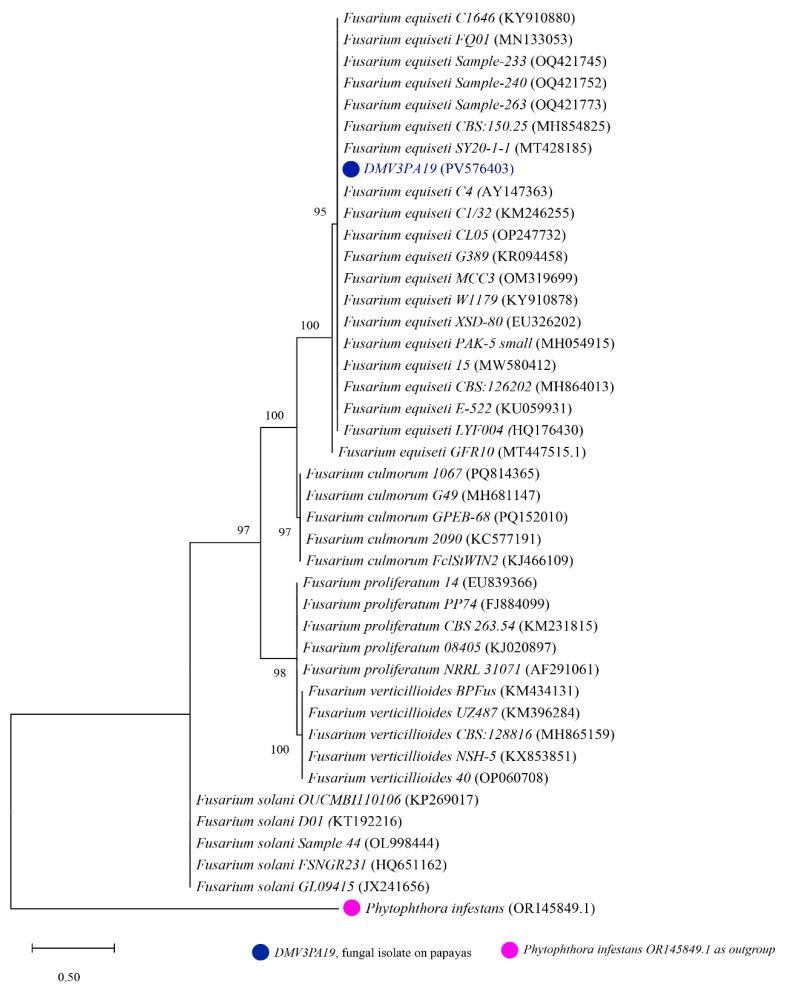
Phylogenetic tree generated from sequences of DMV3 isolate and other reference strains of *Fusarium* spp. with *Phytophthora infestans* (OR145849.1) used as an outgroup. The DMV2 isolate from this study is shown in blue.

**Figure 6:**
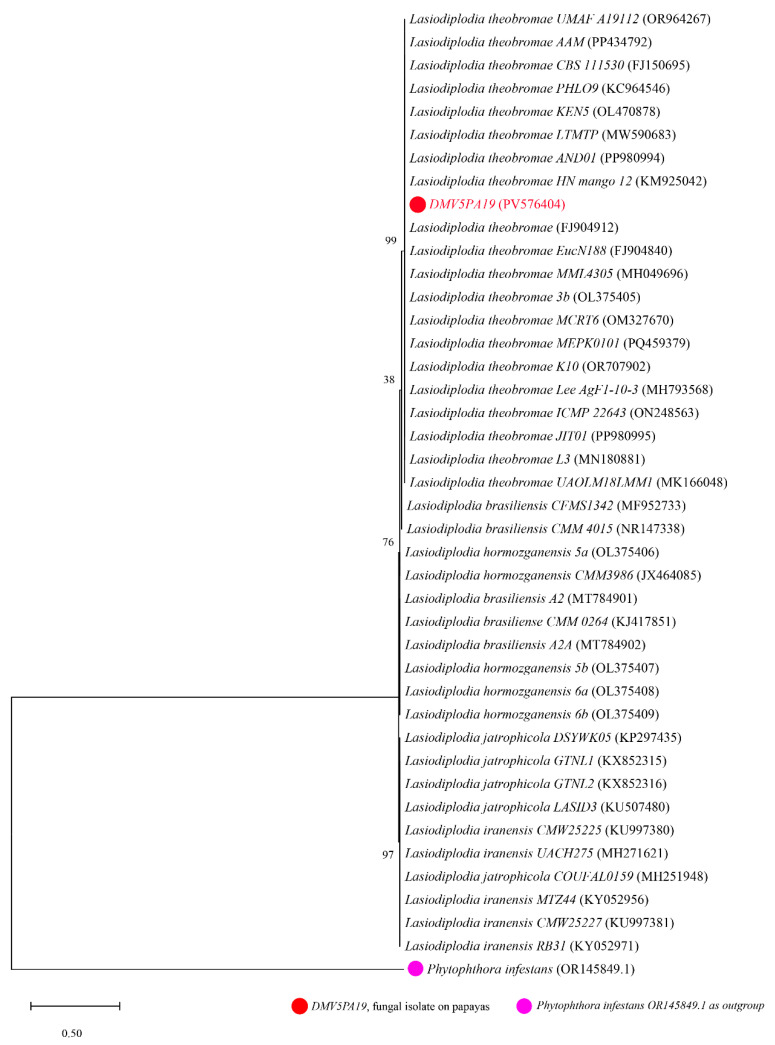
Phylogenetic tree generated from sequences of DMV5 isolate and other reference strains of *Lasiodiplodia* spp. with *Phytophthora infestans* (OR145849.1) used as an outgroup. The DMV2 isolate from this study is shown in red.

For each isolate, 41 nucleotide sequences were analyzed, of which 40 were obtained from GENBANK and one corresponding to one of our isolates. The phylogenetic trees generated are presented in Figure 4, Figure 5 and Figure 6. The phylogenetic tree shown in Figure 4 was constructed using sequences from the DMV2 isolate and other reference strains of *Colletotrichum* spp. The tree is divided into five monophyletic groups. The DMV2 isolate clusters in the same clade as the *C. gloeosporioides* species associated with GenBank accession numbers HQ645073, FJ940734, KJ632401, MT597824, and KU662388, leading to its identification as *C. gloeosporioides*. The other clades correspond to *C. musae*, *C. fructicola*, *C. capsici*, and *C. falcatum* species.

The phylogenetic tree in Figure 5 illustrates the relationship between isolate DMV3 and other reference strains of *Fusarium* spp. The tree is divided into five well-defined clades, each corresponding to different species within the *Fusarium* genus. The DMV3 isolate clusters within the same clade as *F. equiseti* strains (accession numbers MT428185, AY147363, MT447515, OQ421773, and KM246255), leading to its identification as *F. equiseti*. The other four clades correspond to *F. solani*, *F. verticillioides*, *F. culmorum*, and *F. proliferatum* species. Notably, this represents the first report of *F. equiseti* as a pathogen of papaya fruits in Cameroon, which should alert researchers and agricultural monitoring services to consider this pathogen as a pest of *Carica papaya* in the region.

In Figure 6, the evolutionary relationship between the DMV5 isolate and *Lasiodiplodia* spp. strains is illustrated. The phylogenetic tree is organized into five clades. The DMV5 isolate forms a monophyletic clade with *Lasiodiplodia theobromae* species (reference numbers: MH049696, KM925042, MN180881, PP980994, and FJ904912 on GenBank), confirming their assignment to the same species. Other species, including *L. brasiliensis*, *L. hormozganensis*, *L. jatrophicola*, and *L. iranensis*, also form their distinct clades.

### 3.4. Influence of Environmental Factors on Mycelial Growth of Papaya Pathogenic Fungi

Once the identity of the etiological fungi associated with papayas *cv* Sunrise solo rot in Cameroon has been determined, understanding their physiological growth characteristics (temperature, pH, [NaOH], and aw) becomes crucial information. This knowledge will enable the development of better-adapted control strategies.

#### 3.4.1. Effect of Temperature on Fungal Mycelial Growth

**Figure 7:**
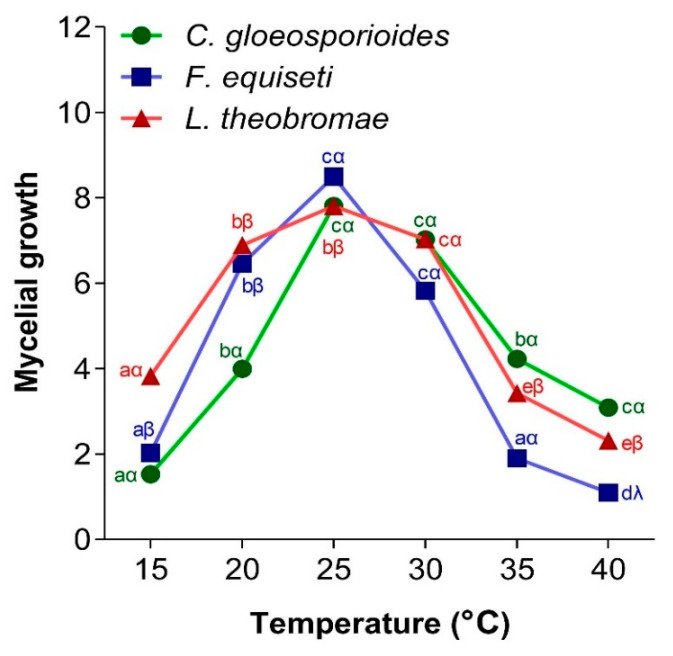
Influence of temperature on mycelial growth of *C. gloeosporioides*, *F. equiseti*, and *L. theobromae.* For a given fungus and according to the different temperature conditions, the points bearing the different letters (a, b, c, d, e) were statistically different according to the Tukey test at *p* < 0.05 (comparison at different temperatures for the germ). For a given temperature, in relation to the three fungi, the points bearing the different signs (α, β, λ) were statistically different according to Tukey’s test at *p* < 0.05 (comparison between the 3 germs).

Temperature is a very important parameter in the mycelial growth of fungi because it affects their growth rate. Figure 7 shows the effect of temperature on the mycelial growth of *C. gloeosporioides*, *F. equiseti*, and *L. theobromae*. It was observed that the three fungi exhibited a bell-shaped growth curve over temperatures ranging from 15 to 40 °C. The optimal temperature for mycelial growth of the three fungi was 25 °C. The lowest mycelial growth of *C. gloeosporioides* was observed at the lowest temperature. Furthermore, the lowest growth of *F. equiseti* and *L. theobromae* was noted at 40 °C.

#### 3.4.2. Effect of pH on Fungal Mycelial Growth

**Figure 8:**
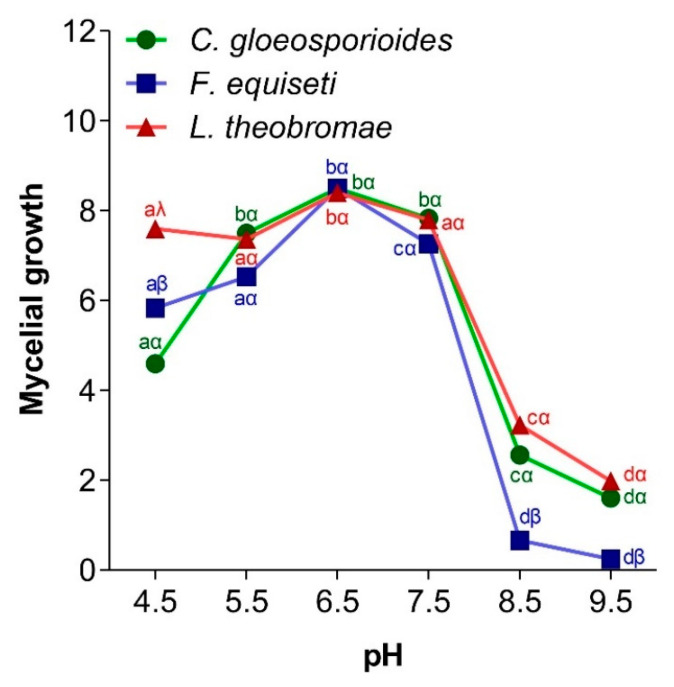
Influence of pH on mycelial growth of *C. gloeosporioides*, *F. equiseti*, and *L. theobromae.* For a given fungus and according to the different pH conditions, the points bearing the different letters (a, b, c, d) were statistically different according to the Tukey test at *p* < 0.05 (comparison at different temperatures for the germ). For a given pH, in relation to the three fungi, the points bearing the different signs (α, β, λ) were statistically different according to Tukey’s test at *p* < 0.05 (comparison between the 3 germs).

The development of pathogenic fungi depends on environmental conditions such as the pH of the medium. Figure 8 shows the influence of pH on the mycelial growth of the three pathogenic fungi of papaya. It appeared that *C. gloeosporioides*, *F. equiseti*, and *L. theobromae* exhibited optimal growth at a pH of 6.5 and minimal growth at a pH of 9.5.

#### 3.4.3. Effect of Salt Stress on Pathogen Growth

**Figure 9:**
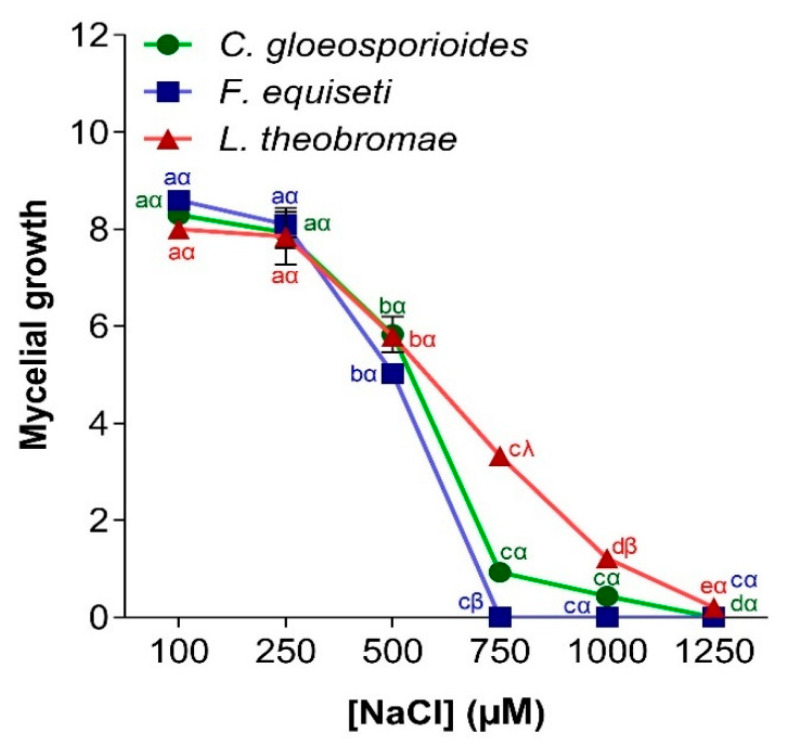
Influence of salt stress on mycelial growth of *C. gloeosporioides*, *F. equiseti*, and *L. theobromae*. For a given fungus and according to the different [NaCl] conditions, the points bearing the different letters (a, b, c, d, e) were statistically different according to the Tukey test at *p* < 0.05 (comparison at different temperatures for the germ). For a given [NaCl], in relation to the three fungi, the points bearing the different signs (α, β, λ) were statistically different according to Tukey’s test at *p* < 0.05 (comparison between the 3 germs).

The salinity of the medium affects the mycelial growth of fungi variably, depending on the species and salt concentration. Figure 9 shows the influence of salt stress on the mycelial development of *C. gloeosporioides*, *F. equiseti*, and *L. theobromae* isolated from papaya fruits. A decreasing curve pattern is observed. The mycelial growth of the three fungi was inversely proportional to the NaCl concentration of the medium. The three fungi had maximum mycelial development at a concentration of 100 µM (with a growth diameter ranging from 8.0 to 8.2 cm). However, beyond 750 µM, the mycelial growth of *F. equiseti* was completely inhibited. The growth of *C. gloeosporioides* and *L. theobromae* was no longer visible at concentrations higher than 1250 µM.

#### 3.4.4. Effect of Water Potential on Pathogen Mycelial Growth

**Figure 10:**
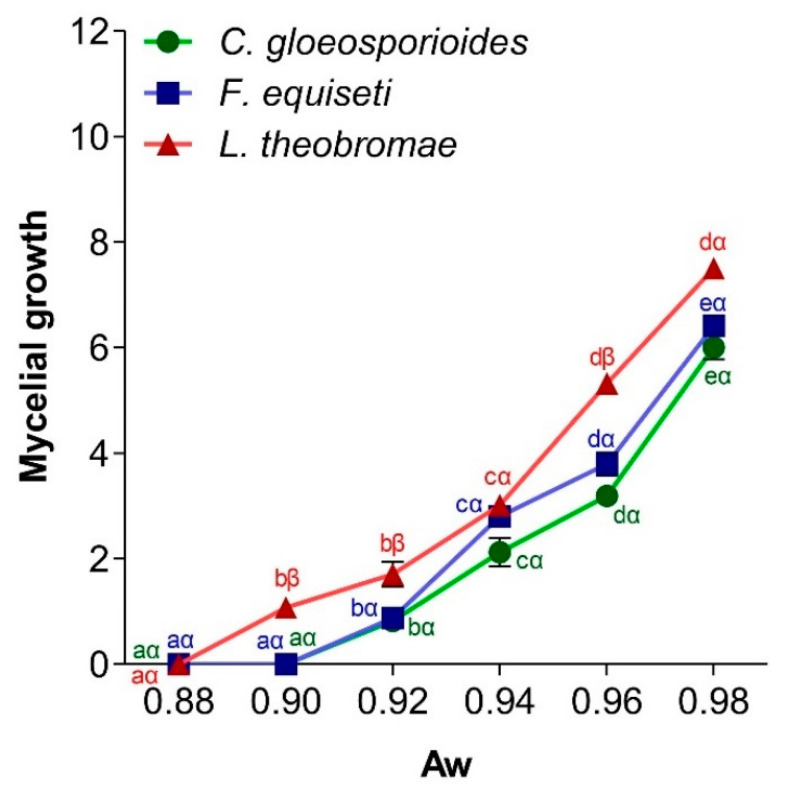
Influence of a_w_ on mycelial growth of *C. gloeosporioides*, *F. equiseti*, and *L. theobromae*. For a given fungus and according to the different a_w_ conditions, the points bearing the different letters (a, b, c, d, e) were statistically different according to the Tukey test at *p* < 0.05 (comparison at different temperatures for the germ). For a given a_w_, concerning the three fungi, the points bearing the different signs (α, β) were statistically different according to Tukey’s test at *p* < 0.05 (comparison between the 3 germs).

The availability of water in an environment is critical for spore hydration, which is essential for initiating germination and mycelium development. Figure 10 demonstrates the impact of medium water activity on the mycelial growth of *C. gloeosporioides*, *F. equiseti*, and *L. theobromae*. The results showed a positively correlated relationship, where the mycelial diameter increases significantly with enhanced water availability. As the water activity of the PDA medium increased, the extent of mycelial growth also increased. Optimal growth for all three fungi occurred at a water activity of 0.98. However, *L. theobromae* exhibited mycelial growth initiation at a relative humidity corresponding to an a_w_ of 0.88, whereas *C. gloeosporioides* and *F. equiseti* showed noticeable growth at an a_w_ of 0.90.

### 3.5. Capacity of Isolated Fungi to Secrete Hydrolytic Enzymes

**Figure 11:**
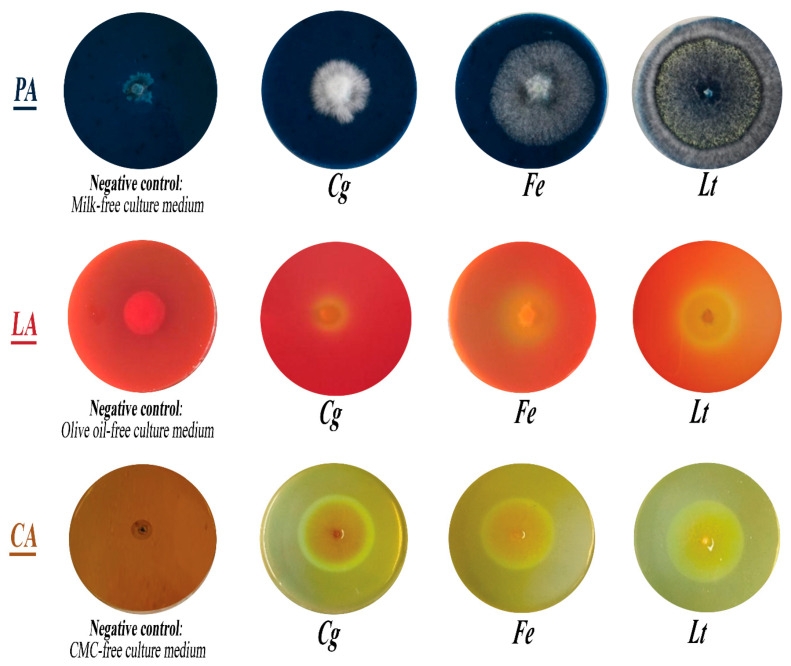
Protease, lipase, and cellulase activities of the three fungi. PA: Protease activity; LA: lipase activity; CA: cellulase activity. *Cg: C. gloeosporioides; Fe: F. equiseti; Lt: L. theobromae;* CMC: Carboxyl Methyl Cellulase.

**Figure 12:**
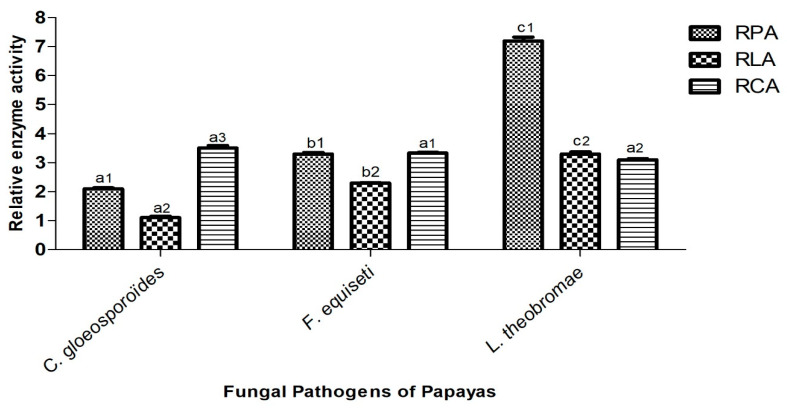
Variation in relative enzymatic activity according to the different pathogenic fungi. RPA: Relative protease activity; RLA: relative lipase activity; RCA: relative cellulase activity. For the same pathogen and different enzymatic activity, the histograms bearing different numbers (1, 2, 3) present a significant difference at *p* ≤ 0.05. For the same enzymatic activity for different fungi, the histograms with different letters (a, b, c) are significantly different at *p* ≤ 0.05 according to Tukey’s test.

The ability of the three isolated fungi (*C. gloeosporioides*, *F. equiseti*, and *L. theobromae*) from papaya *cv* Sunrise solo to secrete hydrolytic enzymes depends not only on the fungal species but also on the type of secreted enzyme. This ability is directly correlated with the relative enzymatic activity (REA), as illustrated in Figure 11 and Figure 12.

The protease activity of *C. gloeosporioides*, *F. equiseti*, and *L. theobromae* was evidenced by their growth on milk agar medium. The fungal colonies exhibited white, cottony mycelial development, surrounded by a transparent halo whose diameter varied depending on the fungal species. This observation highlighted the ability of these fungi to secrete proteases capable of hydrolyzing the skim milk present in the medium. Among the tested species, *L. theobromae* demonstrated the highest relative protease activity (RPA), with a halo diameter of 7.2 cm, followed by *F. equiseti* with 3.3 cm, and *C. gloeosporioides* with an RPA of 2.1 cm.

The lipase production potential of *C. gloeosporioides*, *F. equiseti*, and *L. theobromae* was evaluated on peptone agar medium supplemented with olive oil. Mycelial growth appeared with a persistent yellow halo, whose diameter varied among fungal species. This observation highlights the ability of these fungi to secrete lipases that facilitate the hydrolysis of olive oil in the medium. The relative lipase activity (RLA), measured as halo diameter, was 1.1 cm for *C. gloeosporioides*, 2.3 cm for *F. equiseti*, and 3.55 cm for *L. theobromae*. Among the three species, *L. theobromae* exhibited the highest RLA.

The cellulase secretion potential of *C. gloeosporioides*, *F. equiseti*, and *L. theobromae* was evaluated using carboxymethyl cellulose (CMC) medium. The fungal growth was characterized by brown, fluffy-textured mycelia surrounded by a clear halo, whose diameter varied among the fungal species. This observation reflects the ability of these fungi to produce cellulase enzymes capable of degrading the CMC substrate in the culture medium. The relative cellulase activity (RCA) of *C. gloeosporioides*, *F. equiseti*, and *L. theobromae* exhibited close values, measuring 3.5 cm, 3.3 cm, and 3.1 cm, respectively. Nonetheless, *C. gloeosporioides* demonstrated the highest RCA among the three species.

## 4. Discussion

In this study, seven fungal isolates (DMV1, DMV2, DMV3, DMV4, DMV5, DMV6, and DMV7) were isolated from cv. Sunrise solo papayas. However, only three fungal isolates (DMV2, DMV3, and DMV5) were identified as the main agents responsible for rot and significant post-harvest losses of Sunrise solo papayas in Cameroon. Pathogenicity testing confirmed that these isolates were indeed associated with diseases such as anthracnose, fusariosis, and soft rot in papayas. The morphological characteristics of these three isolates were similar to those of microorganisms isolated by Nuangmek et al. [55] in Thailand, Gnanesh et al. [56] in India, Hu et al. [57] in China, and Helal et al. [58] in Bangladesh, who identified fungal isolates, such as the *Colletotrichum*, *Fusarium*, and *Lasiodiplodia* genera, as pathogens of various crops, particularly *Carica papaya* fruits. Gómez et al. [59] and Pacheco-Esteva et al. [60] established that plant pathogenic fungi belonging to *Colletotrichum*, *Fusarium*, and *Lasiodiplodia* genera were the main agents responsible for post-harvest rot of papaya fruit. The fact that papaya is such a popular substrate for these fungal microorganisms is undoubtedly due to its high water content, considering that water is the major and essential element for microbial development.

Phylogenetic analysis elucidated the evolutionary relationships between pathogenic species isolated from papaya in this study and related strains documented in the NCBI. Across the three generated trees, nodal bootstrap values (99, 100) indicate high statistical reliability of branches, demonstrating robust relationships among species. This underscores the critical role of precise molecular methods for accurate pathogen identification. The analysis revealed genetic diversity within the *Colletotrichum*, *Fusarium*, and *Lasiodiplodia* genera, with clear genetic delineation of multiple species. However, the evolutionary proximity of certain genus/species suggests potential shared pathogenicity genes, particularly those encoding plant cell wall-degrading enzymes or effectors, as well as overlapping host ranges and similar infection mechanisms. Numerous phytopathological studies have demonstrated the direct involvement of these three fungal species as pathogens of papaya and several other fruits. Ghaljaei et al. [61] identified *L. theobromae* as one of the most devastating pathogens of papaya in northeastern Brazil. Research conducted by Karunamoorthy et al. [22] revealed that *L. theobromae* (GenBank accession No. MN335222.1) is responsible for 40% of postharvest losses of papaya in Malaysia, with dark lesions and white rot symptoms. A study performed by José et al. [62] identified *L. theobromae* as the primary etiological agent of jackfruit wilt and gummosis in the Philippines and Brazil. Concerning *C. gloeosporioides*, it has been reported as a papaya fruit pathogen in the United States and Brazil by Sarkhosh et al. [12] and Netto et al. [63], respectively. Rayón et al. [64] identified *C. gloeosporioides* as the most virulent foodborne pathogen causing papaya anthracnose in Mexico. Li et al. [65] described *C. gloeosporioides* (strain Cs GQHZJ19, GenBank accession) as a serious threat to strawberry cultivation in China. Chutima [66] and Chen et al. [67] revealed that anthracnose due to *C. gloeosporioides* is one of the most devastating diseases affecting mangoes in Thailand and strawberries in China, resulting in major losses due to rotting during storage. Regarding *F. equiseti*, multiple studies have documented its role as a rot agent in various crops. Karunamoorthy et al. [22] identified for the first time *F. equiseti* (No. MN335223.1 GenBank accession) as a pathogen of papaya fruit in Malaysia. In India, Gupta et al. [68] reported that 60% of papaya losses were attributable to *Fusarium* spp. infections. However, *F. equiseti* has been implicated in watermelon rot in Malaysia, chili wilt in the northern Himalayas, and durum wheat damping-off in Algeria, as documented by Rahman et al. [18], Hami et al. [23], and Bencheikh et al. [24], respectively. The pronounced involvement of *C. gloeosporioides*, *F. equiseti*, and *L. theobromae* in fruit decay may arise from their ability to persist latently in host tissues during growth and induce persistent rot during maturation. Moreover, the extensive global distribution of these fungi as pathogens of diverse hosts may be attributed to key factors such as their high genetic variability, intrinsic strain variability, and their ability to adapt to diverse environments. It has been recognized that fungal pathogenicity is highly dependent on the strain–host–environment interaction. Accurate identification of the fungi responsible for papaya rot in Cameroon is essential for precisely determining the type of phytopathogenic fungus involved in a disease, understanding its infection mechanisms, and elucidating its virulence factors. This knowledge is necessary for evaluating potential disease risks, developing effective preventive and surveillance strategies, such as crop rotation, deploying resistant cultivars, and managing environmental conditions conducive to fungal growth.

The correlation between fungal identification and knowledge of their environmental parameters facilitates the elucidation of the infection process, refinement of epidemiological models, customization of interventions according to specific agroclimatic contexts, and development of more targeted control strategies [69,70,71].

Temperature has a significant influence on the metabolic processes of living organisms and affects almost all aspects of the growth and development of microorganisms. It is an essential parameter in the development of phytopathogenic fungi. Each fungal strain has an optimal temperature at which its growth and the expression of diseases it induces are maximal [45]. In the present study, *C. gloeosporioides*, *F. equiseti*, and *L. theobromae* showed maximum mycelial growth at 25 °C. The maximum mycelial growth, observed at a temperature of 25 °C, could be explained by the adaptation of these fungi to the temperature conditions of the Njombé-Penja locality. Indeed, papaya fruits are stored at ambient temperature in the locality, and this temperature generally fluctuates between 24 and 26 °C, with a regular average of 25 °C throughout the year. Similar results were obtained by Estrada et al. [71] who found that the most favorable temperature for the growth of *C. gloeosporioides* isolated from mangoes in the Philippines is 25 °C. Also, Sajili et al. [70] reported maximum mycelial growth between 20 and 30 °C for strains of *C. gloeosporioides* isolated from papaya in Malaysia. Li et al. [65] demonstrated that the *C. gloeosporioides* species responsible for strawberry rot in China could grow at temperatures ranging from 10 to 26 °C.

Regarding the *F. equiseti* species, the work of Palmero et al. [72] showed that the mycelial growth and pathogenicity of seven strains of *F. equiseti* isolated from the soil of marine bottoms were maximal at 25 °C. Yan and Nelson [73] demonstrated that fungal species of the *Fusarium* genus responsible for the development of soybean disease have a maximum mycelial growth around 24 °C. In addition, the work of Almiman [74], responsible for most plant deterioration in fields and storage warehouses, caused more pronounced symptoms of wilting and leaf spots at a temperature of 25 °C. A study conducted in Cameroon demonstrated that the mycelial growth rate and conidial concentration of *Lasiodiplodia theobromae* isolated from *Ricinodendron heudelotii* seeds increased with rising temperature, reaching an optimum at 23 °C [75]. Although maximum growth is obtained at 25 °C, the three fungi identified in this study had noticeable mycelial growth at temperatures ranging from 15 to 40 °C. However, Félix et al. [76] showed that phytopathogenic fungi, such as *L. theobromae*, which infect different hosts, have a strong capacity to adapt to different environments and grow over a wide range of temperatures (9–39 °C). Indeed, the wide range of temperatures at which *Colletotrichum gloeosporioides*, *Fusarium equiseti*, and *Lasiodiplodia theobromae* can grow and sporulate indicates that they possess a high level of genotypic plasticity that enables them to survive and proliferate in a variety of atmospheric conditions. Although the optimal mycelial growth of *Colletotrichum gloeosporioides*, *Fusarium equiseti*, and *Lasiodiplodia theobromae* was observed at 25 °C in this study, these three fungi had a notable growth capacity at temperatures ranging from 15 to 40 °C. Indeed, the wide range of temperatures at which these three fungi grew and sporulated could be explained by their high level of genetic plasticity, which allows them to survive and proliferate in various atmospheric conditions.

Environmental pH significantly affects fungal development because mycelial growth and conidial germination are limited to a certain pH range for each species. In this study, *Colletotrichum gloeosporioides*, *Fusarium equiseti*, and *Lasiodiplodia theobromae* grew at a pH range of 4.5 to 9.5, with maximum growth at pH 6.5. These results were similar to those of Sajili et al. [70], where after subjecting strains of *C. gloeosporioides* isolated from *Carica papaya* fruit in Malaysia to different pH, they found maximum growth at pH 6.5. The study conducted by Kannan and Dhivya [45], on the influence of pH on the radial growth of *C. gloeosporioides* responsible for mango anthracnose in India, showed that, among the different pH tested, pH 7.0 proved to be the most favorable for good radial growth (69 mm), followed by pH 6.0 (56 mm). The research carried out by Zhao et al. [77] demonstrated that the optimum pH for the growth of *F. equiseti* isolated on crops in China was between 4.46 and 5.11. Also, the work of Chakrapani et al. [78] showed that pathogens of the *Fusarium* genus isolated from peas (*Pisum sativum *L.) could grow under pH conditions ranging from 3.0 to 9.0, with higher mycelial growth at pH 5.0. Concerning *L. theobromae*, Febbiyanti et al. [79] showed that this fungus, responsible for stem canker on rubber trees, grew at a pH of 4.0 up to a pH of 9, with an optimum observed at pH 7.0. Also, Latha et al. [80] demonstrated that *L. theobromae* isolated from *Jatropha curcas* (Jatropha) grows up to a pH of 9.0, with optimal growth observed at pH 7.0. The ability of *C. gloeosporioides*, *F. equiseti*, and *Lasiodiplodia theobromae* to grow under different pH conditions can be explained by the genetic diversity of the different morphotypes of the same fungal genus, which tolerate more or less acidic and/or basic conditions. It may also be linked to the nature of the preferred substrate that the fungi colonize [81].

The salinity of the medium is one of the environmental factors that conditions the development and degree of infection of microorganisms. NaCl can inhibit or promote the mycelial development of phytopathogenic fungi, depending on its concentration in the medium [82]. In this study, *Colletotrichum gloeosporioides*, *Fusarium equiseti*, and *Lasiodiplodia theobromae* grew on PDA medium at NaCl concentrations ranging from 100 to 1250 μM. Mycelial growth was highest at a NaCl concentration of 100 µM. These results corroborated the work of Palmero et al. [72], which showed that after studying the effect of saline media on the pathogenicity and growth of *F. equiseti* isolated from host plants in Spain, a slowdown in the growth of these strains was observed in media with a NaCl concentration equal to 1000 μM. Also, Feng et al. [83] investigated the effect of salt stress on the growth of the *F. equiseti* (Z7 strain), pointing out that it grew at NaCl concentrations ≥ 1000 µM. Furthermore, Li et al. [84] showed that the pathogenicity of *C. gloeosporioides* could be observed on PDA medium containing 1.2 M NaCl. In addition, the study carried out by González et al. [85] showed that *C. gloeosporioides*, responsible for avocado (*Persea americana*) rot in Mexico, showed visible radial growth on PDA medium supplemented with 0.85% NaCl. Investigations by Gunamalai et al. [86] showed that the development of *L. theobromae* was not affected in media containing 4% NaCl. Indeed, the ability of *C. gloeosporioides*, *F. equiseti*, and *L. theobromae* to thrive in environments with diverse salinity levels may be attributed to the tolerance phenomenon acquired, facilitated by their capacity for metabolic and physiological adaptations.

Water is the most important substrate, essential for the development of all living organisms. Its availability, therefore, influences the growth of phytopathogenic fungi and their virulence. In this study, *Colletotrichum gloeosporioides*, *Fusarium equiseti*, and *Lasiodiplodia theobromae* were grown on PDA media with water activities ranging from 0.88 to 0.98. The fastest mycelial growth was observed in the PDA medium with a water activity (aw) of 0.98. Sanzo-Miró et al. [87], in their work on the impact of environmental factors on the growth of *Colletotrichum* species strains isolated from potato tubers in the UK, showed that these strains grew better in media with a water activity of 0.995. Similarly, Almiman [74] demonstrated that the best mycelial growth of *F. equiseti* and *L. theobromae* on PDA medium was achieved with an aw of 0.995. Environments with high relative humidity and high water activity (aw) are favorable for the development of *C. gloeosporioides*, *F. equiseti*, and *L. theobromae* because they use available water molecules to carry out all the biological processes necessary for their growth.

Phytopathogenic fungi are known to produce several types of enzymes, generally involved in the degradation of the plant cell wall, and to induce pathogenesis. In the present study, the capacity of *Colletotrichum gloeosporioides*, *Fusarium equiseti*, and *Lasiodiplodia theobromae* to use cellulose, olive oil, and skim milk as nutritional substrates suggested that they secreted cellulases, lipases, and proteases, visible through growth halos on specific media. The ability to produce these enzymes gave these phytopathogens cellulolytic, lipolytic, and proteolytic activity [88,89]. These results were similar to those of Félix et al. [30] who showed that *L. theobromae* strains isolated from *Vitis vinifera* in Peru (LA-SOL3, LA-SV1 and LA-MA-1) produced amylases, caseinases, cellulases, lipases, and pectinases on specific media at 25 °C. Similarly, Rodríguez-Gálvez et al. [90] revealed that 25 species of *Lasiodiplodia* associated with mango rot used cellulase as an enzyme to induce their pathogenicity. Additionally, Huang et al. [91] demonstrated the ability of *C. gloeosporioides* to secrete three lytic enzymes (cellulose, β-1,3-glucanase, and protease) involved in mango degradation in China. Furthermore, Jat et al. [92] showed that anthracnose caused by *C. gloeosporioides* on bananas in India was due to the secretion of polygalacturonase and cellulase demonstrated in vitro. Cruz-Davila et al. [93] also demonstrated the ability of *Fusarium* species to produce xylanases and cellulases when grown on wheat bran. However, Alvarez-Navarrete et al. [94] highlighted the potential of *Fusarium solani* to produce interesting holocellulolytic activity in a growth medium where corn stalk was the sole carbon source. Many authors have shown that fungal species are capable of producing a multiplicity of extracellular enzymes, which confer a strong contaminating power [76,95]. These hydrolytic enzymes act simultaneously to convert the complex cell–matrix of fruits into a simple molecule [86]. They are therefore considered biochemical enzymes used by *Colletotrichum gloeosporioides*, *Fusarium equiseti*, and *Lasiodiplodia theobromae* to reach and degrade papaya tissues. Cellulases could be involved in the degradation of the pectocellulosic wall and lipases in the digestion of the membrane. The virulence of these fungi would thus be associated with the hydrolytic enzymes they secrete during the invasion of food tissue [76].

## 5. Conclusions

This study on identification and characterization of fungi associated with the deterioration of papaya fruits (*Carica papaya*) in Njombé-Penja, Cameroon, led to the identification of *C. gloeosporioides* as the causal agent of black spot rot (anthracnose), *F. equiseti* as responsible for white rot (fusariosis), and *L. theobromae* as the cause of soft rot. In this study, *Fusarium equiseti* was identified for the first time as a pathogen of papaya fruits from Cameroon. The optimal growth conditions for these three fungi were 25 °C, pH 6.5, [NaCl] equal to 100 µM, and an a_w_ of 0.98. *C. gloeosporioides*, *F. equiseti*, and *L. theobromae* species secrete hydrolytic enzymes such as cellulases, proteases, and lipases, which they use as lytic enzymes to degrade papaya tissues. These phytopathogenic fungi deteriorate the quality of papayas after harvest and reduce their availability in Cameroon. Identifying these fungi and understanding their environmental growth conditions is key information for taking preventive measures in agricultural practices. Future work should assess seasonal effects on fungal prevalence and optimize papaya disease management by combining control methods (biological, chemical, and genetic) while minimizing the impact on the environment.

## Figures and Tables

**Table 1:** Characteristics of the used primers according to White et al. [40].

Primers	Sequences (5′-3′)	Length (Pb)	Tm (°C)	GC (%)
ITS-1	TCCGTTCCTCAACCAGCGG	19	53	63
ITS-4	TCCTCCGCTTATTGATATGC	20	50	45

ITS: Internal transcribed spacer, Pb: base pairs, Tm: average melting temperature.

**Table 2:** Surface and depth of necrosis induced by pathogenic isolates on papayas.

DMV2		DMV3		DMV5	
Surface (cm)	Depth (cm)	Surface (cm)	Depth (cm)	Surface (cm)	Depth (cm)
6.2 ± 0.31 ^1^	1.1 ± 0.13 ^α^	4 ± 0.11 ^2^	0.97 ± 0.02 ^α^	7.4 ± 0.51 ^3^	2.1 ± 0.3 ^β^

Mean surface of necroses bearing the same numbers (^1, 2, 3^) are not significantly different according to Tukey’s test (*p* ≤ 0.05). Mean necrotic depths bearing the same signs (^α, β^) are not significantly different according to Tukey’s test at (*p* ≤ 0.05).

**Table 3:** Macroscopic and microscopic characteristics of DMV2, DMV3, and DMV5.

Isolates	DMV2	DMV3	DMV5	
	Colony shape	Circular	Irregular	Circular
	Relief of the colony	Convex	Bomb	Flat
	Allure of contours	Loop	Wavy	Regular
	Colony surface	Rough	Dense	Smooth
	Mycelium color	White to orange-brown	White	White to light-cream
	Colony Texture	Wrinkled	Cottony	Fluffy
	Consistency	Dried	Mucosa	Creamy
	Opacity	Opaque	Opaque	Translucent
	Growth speed	14.1 mm/day	13 mm/day	44 mm/day
	Type of thallus	No Septa	Septa	Septa
	Shape of conidia	Elongated	Fusiform	Ovoid
	Size of conidia	14.3 µm	18.7 μm	22 μm
	Load of conidia	Mean	Strong	Mean

**Table 4:** Number of base pairs, accession numbers, coverage rate, and percentage similarity of fungal isolates to NCBI reference strains.

Isolates	NCBI AN. of Isolates	BP	NCBI AN. of Reference Strains	Banket (%)	Similarity (%)	Species
DMV2	PV576402	580	FJ940734	100	100	*Colletotrichum gloeosporioides*
			KJ632401	97	100	
			MT597824	95	99.46	
DMV3	PV576403	555	MT447515	99	99.46	*Fusarium equiseti*
			KM246255	99	99.46	
			MW580412	99	99.46	
DMV5	PV576404	555	MH049696	99	99.82	*Lasiodiplodia theobromae*
			MN180881	99	99.64	
			MH793568	99	99.64	

BP: Base pairs; AN: accession numbers.

## Data Availability

All data are included in the manuscript. The DNA sequences of the various fungi identified in this study are available on request from the corresponding authors.

## Supplementary Materials

The following supporting information can be downloaded at: https://www.mdpi.com/article/10.3390/jof11050385/s1, Table S1: Details of the 120 sequences used in the phylogenetic tree.
