# Supplementary material for: Morphological, Physiological, Biochemical, and Molecular Characterization of Fungal Species Associated with Papaya Rot in Cameroon
Source: J Fungi (Basel). 2025 May 17;11(5):385. doi: 10.3390/jof11050385 (PMC12112994; doi:10.3390/jof11050385)
Supplement: Supplementary file 1 [file jof-11-00385-s001.zip › jof-3595005-supplementary.pdf]

Table S1: Details of the 120 sequences used in the phylogenetic tree.

		Taxon	Culture/Strain code	Genbank number	Base pairs
COLLETOTRICHUM GENUS	1	*Colletotrichum gloeosporioides*	Cg-03	FJ940734	580
	2	*Colletotrichum gloeosporioides*	CG03	KJ632401	564
	3	*Colletotrichum gloeosporioides*	MEBP0008	MT597824	582
	4	*Colletotrichum gloeosporioides*	SDAU-08-77	FJ550213	574
	5	*Colletotrichum gloeosporioides*	HBwh-2	HQ645073	579
	6	*Colletotrichum gloeosporioides*	CgloTIN03	KF053199	579
	7	*Colletotrichum gloeosporioides*	FD	KX022496	583
	8	*Colletotrichum gloeosporioides*	MEBP 0131	MT672564	565
	9	*Colletotrichum gloeosporioides*	Ry_Wj03	PP808952	576
	10	*Colletotrichum gloeosporioides*	SY	KX022506	587
	11	*Colletotrichum gloeosporioides*	CCGHN03	GQ424104	575
	12	*Colletotrichum gloeosporioides*	79CA/S	GU066652	574
	13	*Colletotrichum gloeosporioides*	CG14	KJ632428	563
	14	*Colletotrichum gloeosporioides*	SA1	KU662388	575
	15	*Colletotrichum gloeosporioides*	Cg-20	HM161642	579
	16	*Colletotrichum gloeosporioides*	C18	KC010549	583
	17	*Colletotrichum gloeosporioides*	CIMAP : 72013	KJ617392	575
	18	*Colletotrichum gloeosporioides*	LB	KX022503	583
	19	*Colletotrichum gloeosporioides*	C-1001	KY474341	574
	20	*Colletotrichum gloeosporioides*	B3159	MT043781	576
	21	*Colletotrichum musae*	ZCL2	KX347473	537
	22	*Colletotrichum musae*	8Ahv	MF171152	537
	23	*Colletotrichum musae*	cm1	PQ289237	536
	24	*Colletotrichum musae*	UNBF25	LC565438	552
	25	*Colletotrichum musae*	LC0962	JN943076	538
	26	*Colletotrichum fructicola*	MST 5M1-1	KJ883597	599
	27	*Colletotrichum fructicola*	yc03	MF540883	613
	28	*Colletotrichum fructicola*	TARI-F217125	MW492609	599
	29	*Colletotrichum fructicola*	MD-1	ON692784	599
	30	*Colletotrichum fructicola*	YCPIC1	OP862812	599
	31	*Colletotrichum capsici*	R11	DQ454016	552
	32	*Colletotrichum capsici*	Skp4	DQ454024	552
	33	*Colletotrichum capsici*	SDAU08-4	EU979361	580
	34	*Colletotrichum capsici*	SL204a	FJ968578	538
	35	*Colletotrichum capsici*	BV14	FJ968579	551
	36	*Colletotrichum falcatum*	CoC671	KP184444	559
	37	*Colletotrichum falcatum*	cf01	KU220959	555
	38	*Colletotrichum falcatum*	cfPAR-3	KP205441	558
	39	*Colletotrichum falcatum*	I-29	MN636346	540
	40	*Colletotrichum falcatum*	RR01	KU220961	556
	1	*Fusarium equiseti*	15	MW580412	558
	2	*Fusarium equiseti*	Sample-263	OQ421773	574
	3	*Fusarium equiseti*	C1/32	KM246255	585
	4	*Fusarium equiseti*	C1646	KY910880	571
	5	*Fusarium equiseti*	CL05	OP247732	571
	6	*Fusarium equiseti*	Sample-240	OQ421752	574
	7	*Fusarium equiseti*	G389	KR094458	564
	8	*Fusarium equiseti*	W1179	KY910878	564

FUSARIUM GENUS	9	*Fusarium equiseti*	C4	AY147363	556
	10	*Fusarium equiseti*	PAK-5 small	MH054915	563
	11	*Fusarium equiseti*	XSD-80	EU326202	573
	12	*Fusarium equiseti*	E-522	KU059931	556
	13	*Fusarium equiseti*	CBS:150.25	MH854825	553
	14	*Fusarium equiseti*	CBS:126202	MH864013	554
	15	*Fusarium equiseti*	FQ01	MN133053	572
	16	*Fusarium equiseti*	SY20-1-1	MT428185	574
	17	*Fusarium equiseti*	GFR10	MT447515.1	585
	18	*Fusarium equiseti*	MCC3	OM319699	562
	19	*Fusarium equiseti*	Sample-233	OQ421745	573
	20	*Fusarium equiseti*	LYF004	HQ176430	542
	21	*Fusarium solani*	Sample 44	OL998444	499
	22	*Fusarium solani*	FSNGR231	HQ651162	523
	23	*Fusarium solani*	GL09415	JX241656	576
	24	*Fusarium solani*	OUCMBI110106	KP269017	567
	25	*Fusarium solani*	D01	KT192216	568
	26	*Fusarium verticillioides*	CBS:128816	MH865159	522
	27	*Fusarium verticillioides*	UZ487	KM396284	570
	28	*Fusarium verticillioides*	BPFus	KM434131	571
	29	*Fusarium verticillioides*	NSH-5	KX853851	532
	30	*Fusarium verticillioides*	40	OP060708	570
	31	*Fusarium culmorum*	2090	KC577191	548
	32	*Fusarium culmorum*	FclStWIN2	KJ466109	535
	33	*Fusarium culmorum*	G49	MH681147	546
	34	*Fusarium culmorum*	GPEB-68	PQ152010	522
	35	*Fusarium culmorum*	1067	PQ814365	549
	36	*Fusarium proliferatum*	NRRL 31071	AF291061	596
	37	*Fusarium proliferatum*	14	EU839366	568
	38	*Fusarium proliferatum*	PP74	FJ884099	506
	39	*Fusarium proliferatum*	08405	KJ020897	585
	40	*Fusarium proliferatum*	CBS 263.54	KM231815	572
LASIODIPLO	1	*Lasiodiplodia theobromae*	MCRT6	OM327670	580
	2	*Lasiodiplodia theobromae*	MML4305	MH049696	590
	3	*Lasiodiplodia theobromae*	AND01	PP980994	565
	4	*Lasiodiplodia theobromae*	L3	MN180881	573
	5	*Lasiodiplodia theobromae*	CBS 111530	FJ150695	668
	6	*Lasiodiplodia theobromae*	UMAF A19112	OR964267	611
	7	*Lasiodiplodia theobromae*	EucN188	FJ904840	582
	8	*Lasiodiplodia theobromae*	Lee AgF1-10-3	MH793568	571
	9	*Lasiodiplodia theobromae*	JIT01	PP980995	555
	10	*Lasiodiplodia theobromae*	MEPK0101	PQ459379	547
	11	*Lasiodiplodia theobromae*	/	FJ904912	580
	12	*Lasiodiplodia theobromae*	PHLO9	KC964546	565
	13	*Lasiodiplodia theobromae*	HN mango 12	KM925042	554
	14	*Lasiodiplodia theobromae*	UAOLM18LMM1	MK166048	546
	15	*Lasiodiplodia theobromae*	3b	OL375405	580
	16	*Lasiodiplodia theobromae*	ICMP 22643	ON248563	580
	17	*Lasiodiplodia theobromae*	AAM	PP434792	583
	18	*Lasiodiplodia theobromae*	LTMTP	MW590683	577
	19	*Lasiodiplodia theobromae*	KEN5	OL470878	562
	20	*Lasiodiplodia theobromae*	K10	OR707902	580

D	21	*Lasiodiplodia brasiliensis*	CMM 4015	NR_147338	546
I	22	*Lasiodiplodia brasiliensis*	CMM 0264	KJ417851	545
A	23	*Lasiodiplodia brasiliensis*	A2	MT784901	553
	24	*Lasiodiplodia brasiliensis*	A2A	MT784902	553
G	25	*Lasiodiplodia brasiliensis*	CFMS1342	MF952733	568
E	26	*Lasiodiplodia hormozganensis*	5a	OL375406	580
N	27	*Lasiodiplodia hormozganensis*	5b	OL375407	580
U	28	*Lasiodiplodia hormozganensis*	6a	OL375408	580
S	29	*Lasiodiplodia hormozganensis*	6b	OL375409	580
	30	*Lasiodiplodia hormozganensis*	CMM3986	JX464085	545
	31	*Lasiodiplodia jatrophicola*	DSYWK05	KP297435	536
	32	*Lasiodiplodia jatrophicola*	LASID3	KU507480	542
	33	*Lasiodiplodia jatrophicola*	COUFAL0159	MH251948	528
	34	*Lasiodiplodia jatrophicola*	GTNL1	KX852315	515
	35	*Lasiodiplodia jatrophicola*	GTNL2 18S	KX852316	515
	36	*Lasiodiplodia iranensis*	CMW25225	KU997380	523
	37	*Lasiodiplodia iranensis*	CMW25227	KU997381	533
	38	*Lasiodiplodia iranensis*	MTZ44	KY052956	526
	39	*Lasiodiplodia iranensis*	RB31	KY052971	582
	40	*Lasiodiplodia iranensis*	UACH275	MH271621	535
